# Optimizing Implementation in Cancer Control (OPTICC): protocol for an implementation science center

**DOI:** 10.1186/s43058-021-00117-w

**Published:** 2021-04-23

**Authors:** Cara C. Lewis, Peggy A. Hannon, Predrag Klasnja, Laura-Mae Baldwin, Rene Hawkes, Janell Blackmer, Ashley Johnson, OPTICC Consortium, represented by Bryan J. Weiner, Paula Blasi, Diana Buist, Allison Cole, Shannon Dorsey, Marlaine Figueroa Gray, Nora B. Henrikson, Rachel Issaka, Salene Jones, Sarah Knerr, Aaron R. Lyon, Lorella Palazzo, Laura Panattoni, Michael D. Pullmann, Leah Tuzzio, Thuy Vu, John Weeks, Rachel Winer

**Affiliations:** 1 Kaiser Permanente Washington Health Research Institute, 1730 Minor Avenue, Suite 1600, Seattle, WA 98101 USA; 2 Department of Health Services, University of Washington, Seattle, WA USA; 3 School of Information, University of Michigan, Ann Arbor Michigan, USA; 4 Department of Family Medicine, University of Washington, Seattle, WA USA; 5 Department of Global Health, University of Washington, Seattle, WA USA; 6 Public Health Sciences Division, Fred Hutch, Seattle, Washington USA; 7 Hutchinson Institute for Cancer Outcomes Research (HICOR), Fred Hutch, Seattle, Washington USA; 8 Clinical Research Division, Fred Hutch, Seattle, Washington USA; 9 School of Public Health, University of Washington, Seattle, Washington USA

**Keywords:** Implementation science, Agile science, Cancer control, Determinants, Mechanisms, Strategies, Optimization, Cancer prevention, Cancer screening

## Abstract

**Background:**

Evidence-based interventions (EBIs) could reduce cervical cancer deaths by 90%, colorectal cancer deaths by 70%, and lung cancer deaths by 95% if widely and effectively implemented in the USA. Yet, EBI implementation, when it occurs, is often suboptimal. This manuscript outlines the protocol for Optimizing Implementation in Cancer Control (OPTICC), a new implementation science center funded as part of the National Cancer Institute Implementation Science Consortium. OPTICC is designed to address three aims. Aim 1 is to develop a research program that supports developing, testing, and refining of innovative, efficient methods for optimizing EBI implementation in cancer control. Aim 2 is to support a diverse implementation laboratory of clinical and community partners to conduct rapid, implementation studies anywhere along the cancer care continuum for a wide range of cancers. Aim 3 is to build implementation science capacity in cancer control by training new investigators, engaging established investigators in cancer-focused implementation science, and contributing to the Implementation Science Consortium in Cancer.

**Methods:**

Three cores serve as OPTICC’s foundation. The Administrative Core plans coordinates and evaluates the Center’s activities and leads its capacity-building efforts. The Implementation Laboratory Core (I-Lab) coordinates a network of diverse clinical and community sites, wherein studies are conducted to optimize EBI implementation, implement cancer control EBIs, and shape the Center’s agenda. The Research Program Core conducts innovative implementation studies, measurement and methods studies, and pilot studies that advance the Center’s theme. A three-stage approach to optimizing EBI implementation is taken—(I) identify and prioritize determinants, (II) match strategies, and (III) optimize strategies—that is informed by a transdisciplinary team of experts leveraging multiphase optimization strategies and criteria, user-centered design, and agile science.

**Discussion:**

OPTICC will develop, test, and refine efficient and economical methods for optimizing EBI implementation by building implementation science capacity in cancer researchers through applications with our I-Lab partners. Once refined, OPTICC will disseminate its methods as toolkits accompanied by massive open online courses, and an interactive website, the latter of which seeks to simultaneously accumulate knowledge across OPTICC studies.

Contributions to the literature

- This manuscript outlines the protocol for the Optimizing Implementation in Cancer Control (OPTICC) Center, which aims to support the development, testing, and refinement of innovative, efficient, and economical implementation science methods.
- OPTICC brings together multiple disciplines and methodological advances to conduct rapid implementation studies across the cancer care continuum for a wide range of cancers in partnership with a diverse laboratory of clinical systems and community organizations.
- This manuscript articulates an approach to capacity building in implementation science that falls along a continuum of immersive to consultative and, if project-driven, utilizing OPTICC-developed and refined methods.

## Optimizing Implementation in Cancer Control (OPTICC): protocol for an implementation science center

The next decade offers an unparalleled opportunity for implementation science to reduce cancer burden for fifteen million people in the USA who will be diagnosed with cancer [1]. Evidence-based interventions (EBIs) could reduce cervical cancer deaths by 90%, colorectal cancer deaths by 70%, and lung cancer deaths by 95% if widely and effectively implemented in the USA [1]. Yet, EBI implementation, when it occurs, is often suboptimal. In “implementation as usual,” implementation strategies are not often matched to important contextual factors; instead, they are selected based on personal preference and organizational routine, for example. Guidance for matching strategies to determinants (i.e., barriers and facilitators; see key terms and definitions in Table 1) is lacking even for established strategies like audit and feedback, which can be carried out in many ways [3, 4]. For implementation science to support optimized EBI implementation, four critical barriers must be overcome: (1) underdeveloped methods for determinant identification and prioritization [5], (2) incomplete knowledge of strategy mechanisms [6, 7], (3) underuse of methods for optimizing strategies [7], and (4) poor measurement of implementation constructs [8, 9].

**Table 1:** Key terms and definitions

Term	Definition
Determinant	Barriers or facilitators of implementing a new clinical practice
Precondition	Factor that is necessary for an implementation mechanism to be activated
Mediator	Intervening variable that may account for the relationship between the implementation strategy and the implementation outcome
Moderator	Factor that increases or decreases the level of influence of an implementation strategy
Mechanism	Basis for an implementation strategy’s effect—processes or events responsible for change produced by strategies. Mechanisms are always mediators, but the reverse is not true [2]
Proximal outcome	Product of the implementation strategy that is realized because of its specific mechanism of action. The most immediate, observable outcome in the causal pathway
Implementation outcome	Outcome that the implementation processes intend to achieve. Not the immediate outcome in the causal pathway

### Underdeveloped methods for determinant identification and prioritization

Settings in which cancer control EBIs are implemented can have dozens of implementation determinants [10, 11], complicating decisions about which to prioritize and target with implementation strategies. Typically, determinants of cancer control EBI implementation are identified by participant interviews, focus groups, or surveys with providers and/or healthcare administrators using general determinants frameworks such as the Consolidated Framework for Implementation Research (CFIR) or the Theoretical Domains Framework [11–18]. These easy-to-use methods for determinant identification can promote consistency and cumulativeness across studies; however, they are subject to the limitations of self-report, including low recognition by participants (insight), low saliency (recall), and low disclosure (social desirability). These methods are also dependent on the psychometric strength of the measures employed, which tends to be low or unknown [19]. Moreover, general determinants frameworks identify general determinants; EBI- or setting-specific determinants may go undetected. Finally, these methods (e.g., surveys, focus groups) often identify more determinants than can be addressed with available resources [11]. Yet methods for prioritizing identified determinants are rarely reported [5]. Those methods that have been reported favor prioritization of “feasible” determinants to address, not necessarily the determinants with greatest potential to undermine implementation [20]. To support optimized EBI implementation in cancer control, advances are needed in the methods for identifying and prioritizing determinants.

### Incomplete knowledge of strategy mechanisms

*Mechanisms* are the processes through which implementation strategies produce their effects [2]. Much like knowing how hammers’ and screwdrivers’ work supports the selection of one tool over the other for specific tasks (e.g., hanging a picture), knowing how strategies work supports effective matching of strategies to determinants. For example, clinical reminders for cancer screening [strategy] are effective in addressing provider habitual behavior [determinant] by providing a cue to action [mechanism] at the point of care [7]. Although strategies have been compiled, labeled, and defined, their mechanisms remain largely unknown [6, 21]. Published systematic reviews of strategy mechanisms in mental health [6] and health care [21] revealed few mechanistic studies and only one empirically supported mechanism. Matching strategies to determinants absent knowledge of mechanisms is largely guesswork, like selecting a tool for a specific task without knowing how any of your tools work. This guesswork is evident in the relative lack of consensus among implementation scientists about which strategies among the 73 described in the Expert Recommendations for Implementing Change compilation best address the 39 potential determinants in the CFIR [22, 23]. More theoretical and empirical work is needed to establish strategy mechanisms to support effective strategy-determinant matching in cancer control EBI implementation.

### Underuse of methods for optimizing strategies

In testing implementation strategies, researchers typically conduct a formative assessment to identify determinants, develop a multi-component strategy thought to address the determinants, pilot the strategy, and evaluate it in a randomized controlled trial (RCT). Well-conducted trials can generate robust evidence about the effectiveness of a multicomponent strategy, as a package, *in the form it took in the evaluation* (e.g., the specific way audit and feedback was conducted). However, this approach has three limitations: (i) The reliance on RCTs for experimental control and the focus on implementation outcomes (e.g., screening rates) limit opportunities to determine if strategy components are addressing identified determinants. (ii) RCTs of multi-component strategies provide limited information about which components drive effects, if all components are needed, how component strategies interact, how strategies should be modified to be more effective, and which combination of strategy components are most cost-effective. (iii) The jump from pilot study to RCT leaves little room for optimizing strategy delivery such as ensuring the most effective and efficient format, source, or dose is used. Thus, multi-component strategies evaluated in expensive, time-consuming RCTs are often suboptimal in their mode of delivery (e.g., in-person versus virtual), their potency to change clinical practice, and their costs to deploy. Moreover, when trials generate null results, as they often do, determining why is nearly impossible. These limitations can be addressed using principles from *agile science* [24, 25], a multidisciplinary method for creating and evaluating interventions through user-centered design and optimization.

### Poor measurement of implementation constructs

To optimize EBI implementation in cancer control, implementers need reliable, valid, pragmatic measures to identify local implementation determinants, assess mechanism activation, and evaluate implementation outcomes. Systematic reviews indicate few such measures exist [9, 26, 27]. Most available measures of implementation constructs have unknown or dubious reliability and validity [9, 26–29]; moreover, many lack the pragmatic features valued by implementers: relevance, brevity, low burden, and actionability [30–32]. Although work to develop better measures to guide implementation research and practice is underway [33–35], more work is needed to address the measurement gap for key implementation constructs.

### Optimizing Implementation in Cancer Control (OPTICC)

*Opt*imizing *I*mplementation in *C*ancer *C*ontrol (P50CA244432) was funded by the National Cancer Institute as one of seven Implementation Science Centers through a one-time strategic request for proposals [36]. OPTICC is a collaboration of the University of Washington (UW), Kaiser Permanente Washington Health Research Institute (KPWHRI), and the Fred Hutchinson Cancer Research Center (FHCRC). OPTICC’s mission is to improve cancer outcomes by supporting optimized EBI implementation in community and clinical settings for a wide range of cancers across the cancer care continuum. OPTICC is guided by three specific aims.

- *Aim 1*. Develop a research program that supports development, testing, and refinement of innovative, efficient, and economical methods for optimizing EBI implementation in cancer control
- *Aim 2*, Support a diverse implementation laboratory of clinical and community partners to conduct rapid, implementation studies anywhere along the cancer care continuum for a wide range of cancers
- *Aim 3*. Build implementation science capacity in cancer control by training new investigators, engaging established investigators in cancer-focused implementation science, and contributing to the Implementation Science Consortium in Cancer

**Fig. 1:**
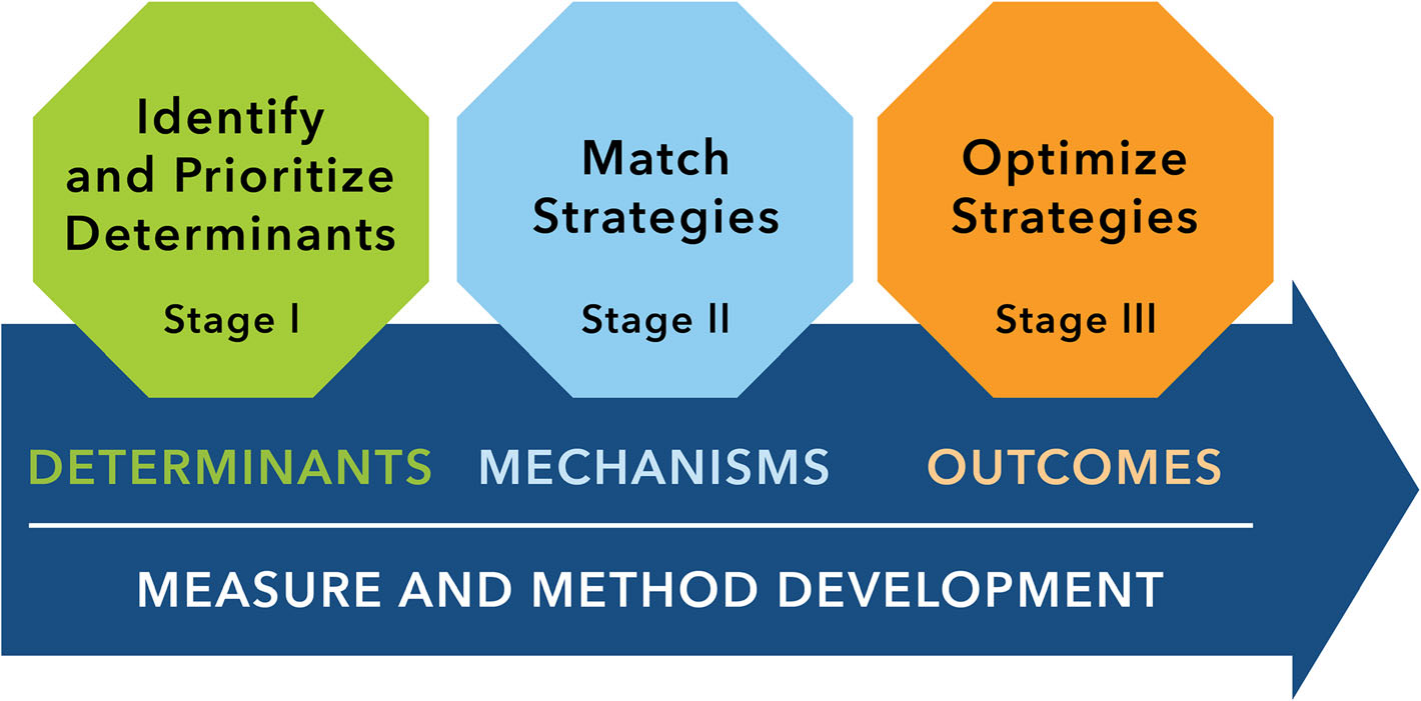
OPTICC stages for optimizing EBI implementation

Three cores support OPTICC’s aims. The Administrative Core plans coordinate and evaluate the Center’s activities and lead its capacity-building efforts. The Implementation Laboratory Core coordinates a network of diverse clinical and community sites to conduct studies to optimize EBI implementation, implement cancer control EBIs, and shape the Center’s agenda. The Research Program Core conducts innovative implementation studies, measurement and methods studies, and pilot studies that advance the Center’s theme of optimizing EBI implementation at any point along the cancer control continuum. A three-stage approach to optimizing EBI implementation is taken—identify and prioritize determinants, match strategies, and optimize strategies (Fig. 1). This protocol manuscript details the partners and methods guiding OPTICC’s work.

## Methods/design

### OPTICC’s Implementation Laboratory

OPTICC established an I-Lab to shape the Center’s research agenda and to partner with OPTICC’s Research Program Core in research to optimize implementation of EBIs in cancer control. The I-Lab is staffed by investigators and staff who conduct cancer implementation research in partnership with clinical and community organizations. We intentionally recruited I-Lab partners that could focus on OPTICC’s optimizing EBI implementation theme at any point along the cancer control continuum. These clinical systems and organizations have a history of research collaboration, and the capacity to grow their collaborations to include implementation research.

The I-Lab includes eight networks and organizations across six states. The I-Lab and its partners represent four main health-related settings: primary care clinics (e.g., federally qualified health clinics, hospital-affiliated clinics, private practices), larger health systems (with hospitals and their affiliated specialty and primary care clinics and services), cancer centers, and health departments (state and local). Table 2 details the characteristics of OPTICC’s I-Lab partners.

**Table 2:** I-Lab partners and their characteristics

Setting	I-Lab partner	Description
Health systems	Kaiser Permanente Washington (KPWA)/Kaiser Permanente Washington Health Research Institute (KPWHRI)	KPWA provides primary, specialty, hospital, home health, and inpatient skilled nursing care on a prepaid (capitation) basis. Kaiser Permanente Washington Health Research Institute (KPWHRI) has a long history of collaborating with the KPWA delivery system to conduct studies embedded in clinical practice.
	Northwest Participant and Clinical Interactions (NW PCI) Network	The NW PCI Network is a collaborative group of clinical and translational research centers, affiliated with medical centers, healthcare systems, clinics and universities spanning the WWAMI region. The NW PCI Network includes primary care and specialty clinics, as well as hospitals.
Primary care	WWAMI region Practice and Research Network (WPRN)^a^	The WPRN is a regional practice-based research network comprising a collaborative group of primary care clinics across 32 organizations in the 5-state WWAMI region. The affiliated practices are diverse, including community health centers, private practices, and university affiliated and government-operated clinics.
	Breast, Cervical, and Colon Health Program (BCCHP) Learning Collaborative	The BCCHP Learning Collaborative is a network of 8 FQHCs in Washington State that receive funding from the Washington State Department of Health (DOH) to implement evidence-based interventions to increase cancer screening rates
Public health		
	Washington Academic Public Health Departments	The WA Academic Health Departments link the University of Washington with Seattle and King County Public Health and the Washington State Department of Health to generate practice-relevant research, assure the utilization of evidence in practice, and grow a competent and evidence-based public health workforce.
Cancer centers	Value in Cancer Care Network (VCCN)	The VCCN is a network of cancer care organizations affiliated with the Hutchinson Institute for Cancer Outcomes Research (HICOR). HICOR was created to bridge research and practice to improve patients’ outcomes by promoting increased and broader use of evidence-based care, and more efficient and effective models of healthcare delivery.
	TriCities Cancer Center	The TriCities Cancer Center is a freestanding non-profit cancer treatment facility that serves the cities of Kennewick, Pasco, Richland, and surrounding rural communities in eastern WA with a mission to provide and coordinate the highest quality, compassionate cancer care.
	Seattle Cancer Care Alliance (SCCA) Network^b^	The SCCA Network Program supports health care organizations in providing community-based oncology services, such as continuing medical education and arranging for local patients to enroll in clinical trials managed by qualified community physicians.

^a^The WWAMI region includes Washington, Wyoming, Alaska, Montana, and Idaho

^b^The SCCA Network includes sites in Washington, Alaska, Montana, Idaho, and Hawaii

### Engaging the I-Lab to shape the OPTICC research agenda

Throughout OPTICC’s life course, we will engage regularly with I-Lab representatives and various members of their organizations and networks. We will ensure that OPTICC’s research agenda includes I-Lab priorities through outreach communications (e.g., a quarterly newsletter), mutual participation in meetings and conferences (i.e., I-Lab representatives will attend OPTICC meetings and I-Lab leads will attend I-Lab partners’ meetings), and annual check-ins with individual I-Lab representatives to assess their organizations’ current cancer control priorities and implementation challenges. This level of engagement will enable OPTICC to (a) create requests for new proposals that address I-Lab partners’ specific cancer control priorities and implementation pain points and (b) foster partnerships between I-Lab members and investigators that result in proposals to do relevant, practice-based implementation research and speed the initial collaboration between investigators and their I-Lab partners.

### OPTICC’s Research Program Core (RPC)

OPTICC’s RPC is staffed by a transdisciplinary group of investigators with expertise in implementation science, clinical psychology, organizational psychology, information science, computer science, sociology, medical anthropology, and public health. The core faculty use team science to develop, test, refine, and disseminate new methods for addressing the four critical barriers via studies taking place in the I-Lab. By design, project leads are supported by RPC core faculty to learn and apply OPTICC methods to build their implementation science capacity via team science. OPTICC approaches optimizing EBI implementation as a three-stage process (Fig. 1). Stage I is identify and prioritize determinants. Providing researchers and implementers with robust, efficient methods for determinant identification and prioritization will enable precise targeting of high-priority problems. Stage II is match strategies. Modeling via causal pathway diagrams (CPDs) to show relationships between strategies, mechanisms, moderators, and outcomes will clarify how strategies function, facilitate effective matching to determinants, and identify the conditions that affect strategy success. Stage III is optimize strategies. Supporting rapid testing in analog (artificially generated experimental conditions) or real-world conditions by testing causal pathways will maximize the accumulation and use of knowledge across projects.

Our focus on implementation strategies and their causal operations is rooted in Collins’s multiphase optimization strategy (MOST [37, 38]) for assessing intervention components and articulating optimization criteria (e.g., per-participant costs) for constructing effective behavioral interventions. In OPTICC, we anticipate that our I-Lab partners will generate questions that span at least seven optimization criteria (Table 3). Based on the state of the science and our partners’ goals, we will help I-Lab partners articulate which optimization criteria to prioritize, especially when they are in conflict, to inform study design and methods. For instance, one of our partners has the goal of increasing HPV home-testing reach using patient outreach materials as an implementation strategy and simultaneously optimize these materials according to patient preference; these criteria are fortunately not in conflict. However, another partner has the goal of increasing practice facilitation impact as an implementation strategy to support colorectal cancer screening while also optimizing efficiency; these criteria may be in conflict, but we have designed a study to test for optimization of each simultaneously (see below). For each of the three optimization stages (Fig. 1), we propose to iterate new methods that can be used independently or be combined depending on which criteria are prioritized in a study. Across methods, we draw on agile science [24, 25], an extension of MOST that emphasizes constructing explicit representations of hypothesized causal pathways that connect strategies to mechanisms, determinants, and outcomes for planning evaluations and organizing evidence. Through agile science, OPTICC draws on user-centered design [39, 40] (UCD) to specifically optimize implementation strategies. UCD is a principled method of technology, EBI, or strategy development that focuses on the needs and desires of end users to create compelling, intuitive, and effective interfaces [41]. A key thread throughout OPTICC is an emphasis on efficient and economical learning, so ineffective ideas are discarded quickly, and additional resources expended only when preliminary evidence indicates that an idea is worth investigating further. Another key thread is an emphasis on usability of evidence for researchers and stakeholders. Below is a summary of our three-stage approach and associated new methods that address limitations of traditional implementation science approaches. Each method will be applied by study leads with support of Research Program Core faculty across OPTICC-funded studies, refined each year, and built into massive open online courses (MOOCs) and toolkits for international dissemination.

**Table 3:** I-Lab partner goals for optimization

Goals	Definitions	Examples
Reach	Maximize number of personnel who can engage	Web-based strategies to limit travel
Preference	Maximize alignment with preferences, values	Strategies that seek provider input
Impact	Maximize fidelity to EBI	EBI practice and feedback
Efficiency	Minimize personnel time	Health information technologies
Cost	Minimize cost	Free surveys for determinant identification
Fit	Maximize alignment with setting infrastructure	Meetings scheduled within workflows
Resources	Maximize use of materials currently available	Strategies built in electronic health record

#### Stage I: Identify and prioritize determinants (Fig. 2)

This stage identifies determinants of implementation success [11] that are active in the specific implementation setting. Strategies not matched to high-priority determinants operating in the implementation setting are unlikely to be effective [5]. Existing methods for this stage have at least four limitations: (I) They typically do not consider relevant determinants identified in the literature. (II) They are subject to issues of recall, bias, and social desirability. (III) They do not sufficiently engage the end user in the EBI prior to assessment. (IV) Approaches to determinant prioritization typically rely on stakeholder ratings of feasibility, among other parameters, of addressing determinants, which may have little to do with impact or import. To address these limitations, we have developed four new, complementary stage I methods.

**Fig. 2:**
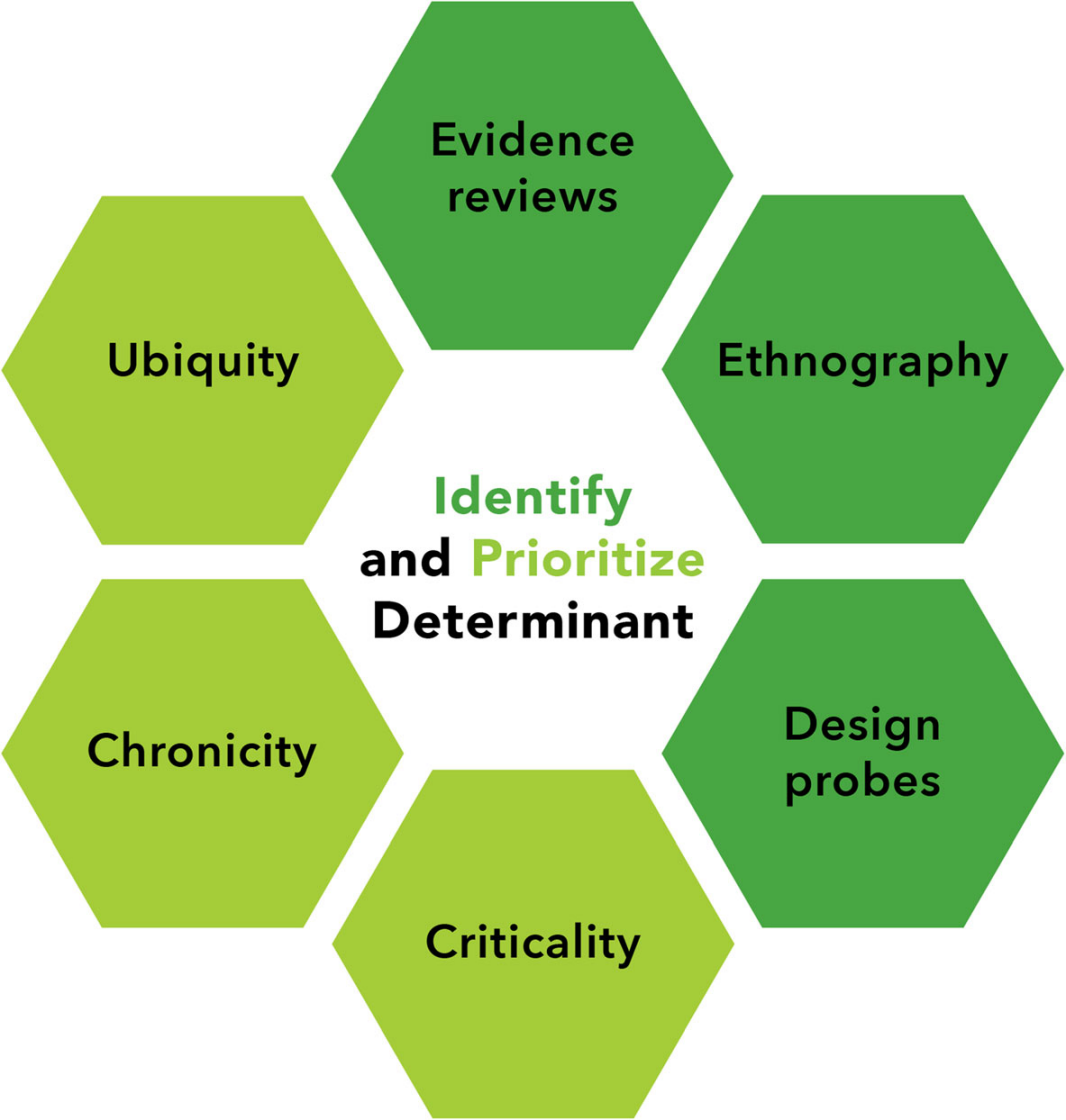
Stage 1: identify and prioritize determinants

First, we will summarize and synthesize research literature on known determinants for implementing EBIs of interest in settings of interest on variants known determinants for implementing the EBI of interest in the settings of interest. Unlike traditional systematic reviews, which can take 12+ months, rapid reviews may be completed in 3 months or less. Rapid evidence reviews are increasingly used in to guide implementation [42–45] in healthcare settings, but they are not typically employed to identify implementation determinants. Implementation Study 1, for example, will ask “What are the known barriers to implementing mailed fecal immunochemical test kit programs in Federally Qualified Health Centers?” The RPC experts will collaborate with project leads and the practice partners to clarify the question and scope each review. Abstraction will focus on identified determinants and any information about timing (i.e., implementation phase), modifiability, frequency, duration, and prevalence. The output will be a list of determinants organized by consumer, provider, team, organization, system, or policy level that will inform observational checklists and interview guides for rapid ethnographic assessment.

Second, rapid ethnographic assessment (REA) will efficiently gather ethnographic data about determinants by seeking to understand people, tasks, and environments from stakeholder perspectives, engaging stakeholders as active participants and applying user-centered approaches to efficiently elicit information. Ethnographic observation will include semi-structured observations and shadowing intended or actual EBI users (e.g., in Implementation Study 1, primary care clinics that implement colorectal cancer screening interventions with and without fidelity), which overcomes self-report biases. Through combined written and audio-recorded field notes, researchers will document activities, interactions, and events (including duration, time, and location); note the setting’s physical layout; and map flows of people, work, and communication. For a range of experiences, ethnographic interviews will be informal during observation and formal through scheduled interactions with key informants. Interviews will be unstructured, descriptive, and ask task-related questions. Researchers will document occurrence or presence of barriers, noting the duration, time, location, and affected persons.

Third, design probes will elicit new and different information from observations and interviews [46–48]. Design probes are user-centered research toolkits with items such as disposable cameras, albums, and illustrated cards. End users are prompted to take pictures, make diary entries, draw maps, or make collages in response to tasks such as “Describe a typical day” or “Describe using [the EBI]”. With design probes, participants have 1 week to observe, reflect on, and report experiences to generate insights, reveal ideas, and illuminate lived experiences as they relate to implementing the EBI [46] (e.g., feelings, attitudes), which overcomes the limitation of assessing stakeholder perceptions in a vacuum. In follow-up interviews, participants will reflect on their engagement with the task. Through memo writing, research team members will analyze the data generated from design probes and interviews to identify new determinants, corroborate determinants discovered via REA, and describe the salience, meaning, and importance of determinants to end users.

Finally, our determinant prioritization methods rely on three criteria: criticality, chronicity, and ubiquity, which overcome the limitations of traditional rating categories that are not clearly linked to impact [49]. *Criticality* is how a determinant affects or likely affects an implementation outcome. Some determinants are prerequisites for outcomes (e.g., EBI awareness). The influence of other determinants on outcomes depends on their potency (e.g., strength of negative attitudes). *Chronicity* is how frequently a determinant occurs in the case of events (e.g., shortages of critical supplies) or persists in the case of states (e.g., unsupportive leadership). *Ubiquity* is how pervasive a determinant is, affecting many EBI implementers. For each identified determinant, granular data generated by the rapid review, rapid ethnographic assessment, and design probes will be organized in a table by these three criteria (criticality, chronicity, and ubiquity) and then independently rated by 3 researchers using a 4-point Likert scale from 0 to 3 (e.g., not at all critical, somewhat critical, critical, necessary) and 3 stakeholders. Priority scores [50–52] and inter-rater agreement will be calculated. The outcome will be a list of determinants ordered by priority scores.

#### Stage II: Match strategies (Fig. 3)

To effectively impact implementation outcomes, strategies must alter prioritized determinants. Drawing on agile science [24, 53], we will develop methods to create CPDs that represent evidence and hypotheses about mechanisms by which implementation strategies impact target determinants and downstream (distal) implementation outcomes. Per Hill [54] and Kazdin [55], we define implementation *mechanisms* as events or processes by which implementation strategies influence implementation outcomes. Our systematic review on mechanisms of implementation found that researchers frequently underspecify (or mis-specify) key factors by labeling them all “determinants,” without declaring the factor’s roles in a strategy’s operation [21]. Moreover, of the 46 studies we identified, none established a mechanism. Also, tests of hypothesized mechanisms often overlooked proximal outcomes and preconditions. As we established previously [7], CPDs would benefit implementation science by (1) driving precision in use of terms for easier comparison of results across studies; (2) articulating hypotheses about the roles of factors that influence implementation strategy functions, enabling explicit testing of these hypotheses; (3) formulating proximal outcomes that can be assessed quickly with rapid analog methods; (4) informing the choice of study designs by clarifying temporal dynamics of represented processes and constraints (e.g., preconditions) that a study must account for [7]; and (5) making evidence more useful and usable. OPTICC’s Research Program Core will support study leads to develop CPDs, which will serve as an organizing structure of our relational database for accumulating knowledge, which is described in more detail in the Discussion. We will create a toolkit for building CPDs with templates, guiding questions, and decision rules. Diagrams will include several key factors (Table 1, Fig. 4): (a) *implementation strategy* intended to influence the target determinant, (b) *mechanism* by which the strategy is hypothesized to affect the determinant, (c) target *determinant*, (d) observable *proximal outcomes* for testing mechanism activation and precursors to implementation outcomes, (e) *preconditions* for the mechanism to be activated and to affect outcome(s), (f) *moderators* (intrapersonal, interpersonal, organizational, etc.) that could impede strategy impact, and (g) *implementation outcomes* that should be altered by determinant changes.

**Fig. 3:**
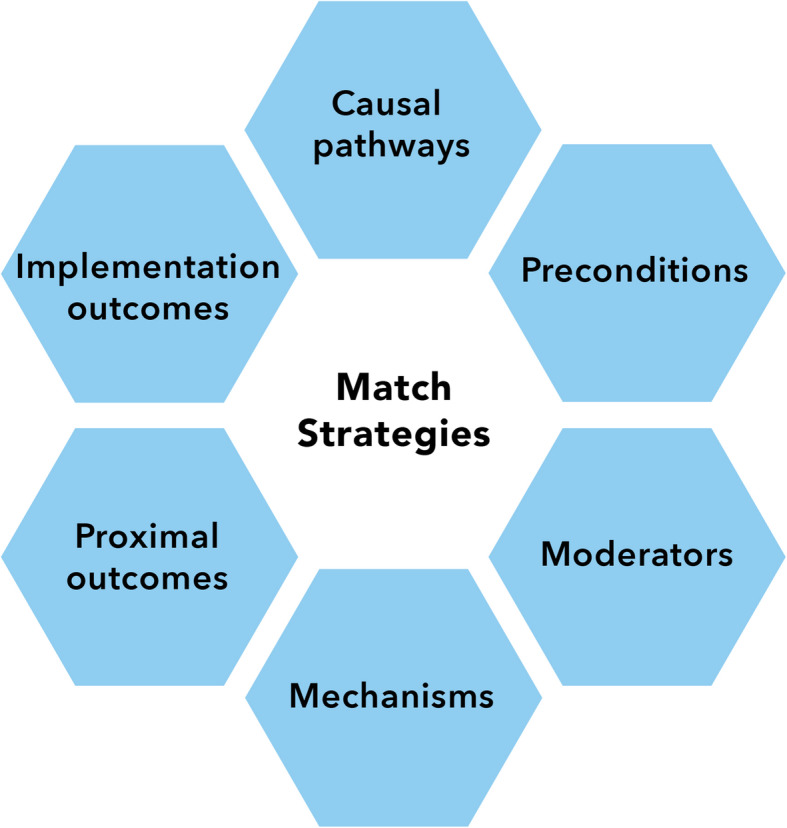
Stage II: match strategies

**Fig. 4:**
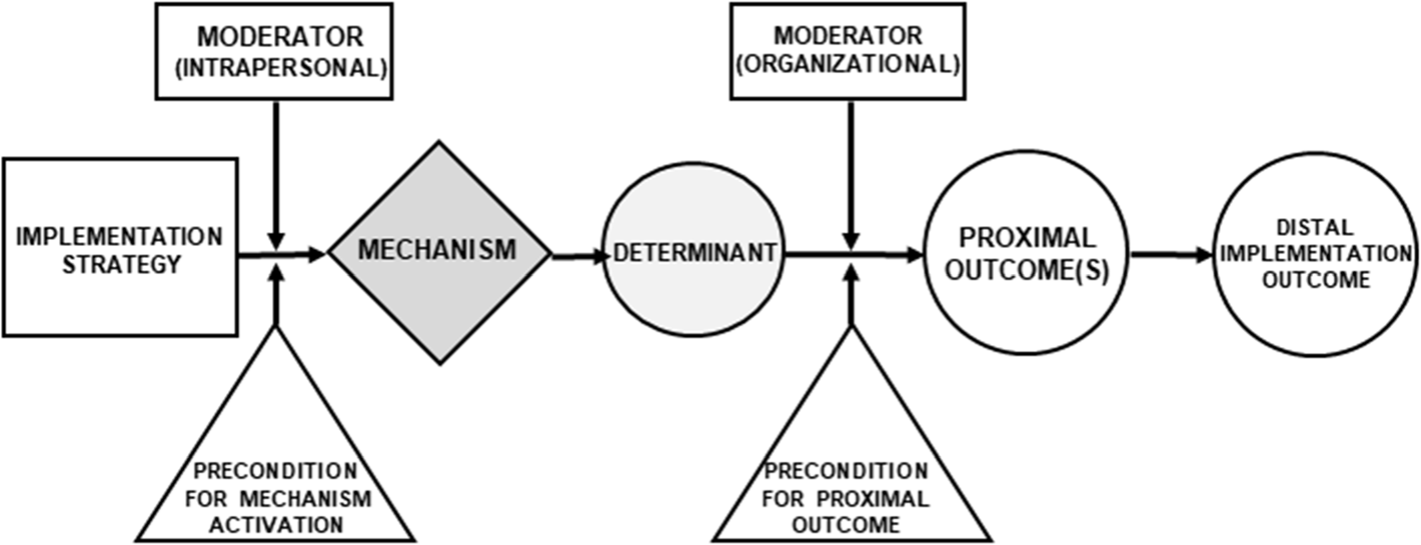
Causal pathway diagram example linear template

CPD construction has five steps that will be elaborated in a methodology paper that includes applications across the initial OPTICC studies. In brief, the steps are as follows: One, teams must select promising strategies to target prioritized determinants. OPTICC suggests that the following inputs should inform strategy selection and differential weight applied to their influence in this order: evidence (i.e., extant literature), plausibility (i.e., a hypothesized strategy-outcome causal chain stands up to logic), feasibility (i.e., the intended site has the capacity to carry out the strategy), and level of analysis (i.e., strategies with direct impact on prioritized determinants are prioritized over those with indirect impact). Two, confirm strategy-determinant alignment by articulating the mechanisms. To this end, articulate concrete operationalizations of the selected strategies as they may take many forms. Three, identify preconditions, which are factors that must be in place for the selected strategy to activate the mechanism. Preconditions are factors that may occur at the intrapersonal, interpersonal, organizational, or system level. Four, identify moderators, which are factors that can amplify or weaken strategy effects and can occur at multiple levels like preconditions. Five, identify proximal outcomes. Too often study teams focus on distal implementation outcomes that may take months or even years to manifest. Identifying proximal outcomes means that observable, measurable, short-term changes can be rapidly detected, which could ultimately save time and money for our partners. If operationalizing the strategy is thought to operate through multiple mechanisms, the same process should be repeated for those mechanisms as well. Once these diagrams are created, these steps should be repeated with different implementation strategy operationalizations to check if different ways of administering the strategy operate through the same mechanisms or if other moderators or preconditions should be considered. For instance, the implementation strategy learning collaboratives could occur in-person or virtually and it would be important to capture CPDs for each to determine if different factors emerge as important. If a strategy can be operationalized in several ways, diagrams should be created for operationalizations being considered for implementation. We acknowledge this represents an overly-simplified, artificially linear representation of implementation, one that we will refine over time, but this process of aligning strategies ➔ mechanisms ➔ determinants ➔ outcomes, and mapping related factors, may still be a practical and useful tool for both researchers and stakeholders.

#### Stage III: Optimize strategies (Fig. 5)

OPTICC will develop and refine methods, guidelines, and decision rules for efficient and economical optimization of implementation strategies, with the objective of helping researchers and stakeholders construct strategies that precisely impact their target determinants. Drawing on MOST and underused experimental methods (Table 4), we will develop guidelines for selecting experimental designs that can efficiently answer key questions at different stages of implementation research and obtain the right level of evidence needed for the primary research question*.* We will prioritize signal testing of individual strategies to identify most promising forms and studies for optimizing blended strategies before testing them in a full-scale confirmatory RCT. Drawing on UCD, we will refine methods for ideation and low-fidelity prototyping to help researchers consider a broader range of alternatives for how an implementation strategy can be operationalized, enabling efficient testing of multiple versions and selecting the version that is most likely to balance effectiveness and burden or cost. We will package developed methods and guidelines as toolkits that will be housed on our publicly available website and will be searchable through the relational database.

**Fig. 5:**
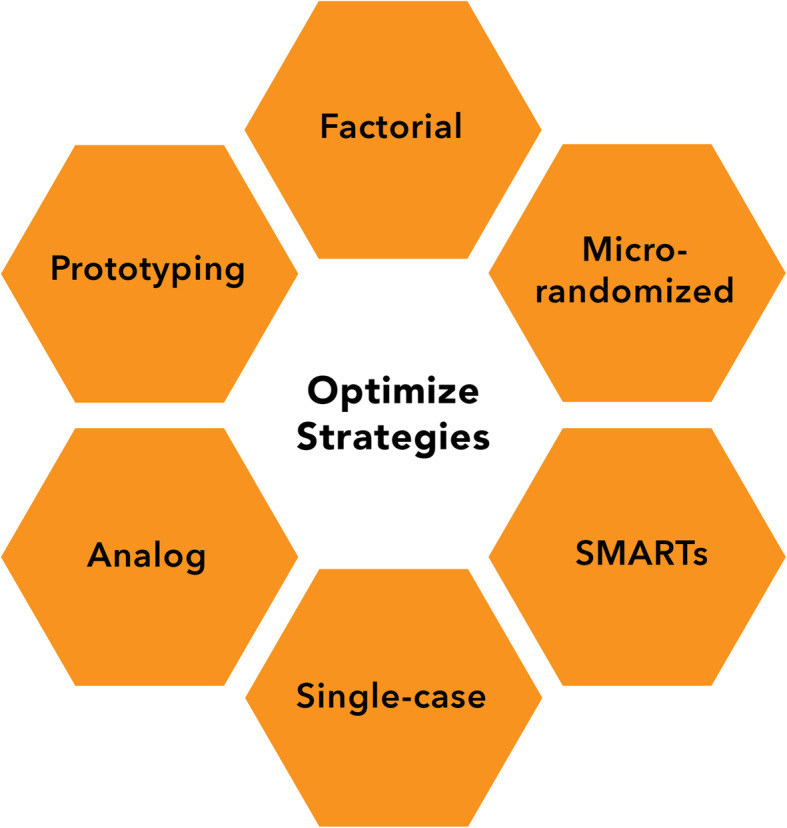
Stage III: optimize strategies

**Table 4:** OPTICC-endorsed designs for efficiently testing and refining strategies for optimizing EBI implementation

Design	Description/benefits
Factorial	Factorial designs are best for optimizing complex strategies [56, 57] because they efficiently screen multiple components for an effect on target outcomes. Each component is a “factor” that can take several “levels” (e.g., yes vs. no; delivery source). Participants are randomized to cells corresponding to different combinations of levels of each factor allowing for analysis of main effects and interactions with fewer participants compared to RCTs.
MRTs	Microrandomized trials (MRTs) evaluate strategy components delivered repeatedly (e.g., automated reminders about assessments). Each time (“decision point”) that a component can be delivered (e.g., patient visit), provision or non-provision of the component is randomized, allowing multiple components to be randomized concurrently. MRTs are a highly efficient design that takes advantage of within-subject and between-subject comparisons to estimate marginal main effects, changes in component effect over time, and moderating effects.
SMARTs	Sequential Multiple Assignment Randomized Trials (SMARTs) optimize adaptive strategies [58, 59] and help researchers determine decision rules for delivering a sequence of strategies that satisfy a set of optimization criteria, usually effectiveness and cost. Participants are initially randomized to two strategies that differ in intensity or cost and at predetermined times, non-responders are re-randomized to another set of strategy options; this can occur multiple times. SMARTs are highly efficient because analyses can use different sample subsets to answer different research questions (e.g., differences between strategies and the optimal way to support non-responders).
SCEDs	Single-Case Experimental Designs (SCEDs) gather evidence about strategy effects by observing changes in outcomes of interest for each participant (or unit, e.g., clinic). SCEDs are inherently within-subject designs with participants acting as their own controls, achieved through sequencing strategy exposures and comparing outcomes for periods when a participant was exposed to those when no strategy was provided. SCED designs include A-B-A-B and multiple baseline approaches. SCEDs require as few as six participants to provide information about effects, making it highly efficient with the low participant requirement making SCEDs promising for preliminary implementation studies in a single clinic [60, 61].

While RCTs provide robust evidence for strategy effectiveness, they do not provide a way to efficiently and rigorously test strategy components [56, 57]. Faced with a similar problem in behavioral intervention science, MOST was developed to help behavioral scientists use a broader range of experimental designs to optimize interventions. The OPTICC Center will leverage these designs to optimize strategies, including: factorial experiments, microrandomized trials (MRTs) [53, 62], sequential multiple assignment randomized trials (SMARTs), and single-case experimental designs (SCEDs) [60, 61]. These designs, described in Table 4 are highly efficient, requiring far fewer participants to test strategy components than a traditional RCT, enabling a range of research questions to be answered in less time and with fewer resources.

### Testing and refining OPTICC’s methods through I-Lab partnered applications

Researchers and implementers can begin work in any of OPTICC’s EBI implementation stages and move forward or backward depending on their optimization goals. A linear progression (stage I ➔ II ➔ III) might be appropriate if researchers or implementers need to clarify critical determinants to select and then test strategies to alter them. Others may have an effective multicomponent strategy that could be optimized by moving backward to mapping strategy components (stage II) and then forward to optimization testing of strategy components before large-scale evaluation or use in clinical or community settings (stage III). OPTICC’s initial studies include those that approach the stages left to right (implementation study 2), right to left (implementation study 1), and those that stay within a single stage (pilot study 2) and those that span two stages (pilot study 1). Measurement development spans the stages; researchers and implementers need robust, useful measures of determinants (stage I), mechanisms (stage II), and outcomes (stage III). Project leads are supported by RPC faculty in their methods application specific to their project work and offered consultation from a national expert to further build their general implementation science capacity.

The first group of I-Lab pilot studies was identified through a competitive process as we wrote our grant proposal for OPTICC. Investigators were encouraged to focus on implementation challenges related to cancer control initiatives. OPTICC investigators evaluated proposals for fit with OPTICC’s methods as well as potential fit with one or more I-Lab partners. Several proposals built on investigators’ established relationships with I-Lab partners, ensuring that there was buy-in from an I-Lab member organization and that our first projects could hit the ground running. What follows is an overview of OPTICC’s initial studies highlighting which stage(s) of EBI implementation they occupy, the optimization goals they are motivated by, and the OPTICC methods applications (Table 5). There will be future open calls for OPTICC studies, which will engage additional I-Lab partners.

**Table 5:** Initial OPTICC-funded projects, their stage, optimization goals, and use of OPTICC methods

Project title	Summary of aims	Stage + methods	Optimization goals
Implementation study 1: ProCRCScreen: increasing colorectal cancer screening in FQHCs through optimized implementation of an evidence-based colorectal cancer screening intervention	1. Examine impact of ProCRCScreen implementation on CRS screening completion 2. Optimize practice facilitation impact on CRC screening rates though feedback on baseline determinants and strategy-determinant alignment	**I**: Rapid Evidence Reviews, Rapid Ethnography, Design Probes, Determinant Prioritization **II**: CPDs **III**: Factorial Design	- Maximize the **efficiency** of practice facilitation using OPTICC methods to conduct a baseline determinant assessment. - Maximize the **impact** of practice facilitation by providing feedback on strategy-determinant alignment.
Implementation study 2: Patient-centered Approach to Tailoring HPV self-sampling for cervical cancer screening (PATH)	1. Develop patient-centered outreach materials addressing uptake determinants to increase home HPV testing 2. Determine if the screening outreach strategy needs to be tailored	**II**: CPDs **III**: Efficient Prototyping, Factorial Design	- Maximize patient **preference** for outreach materials. - Maximize **reach** of home HPV testing through tailoring outreach materials.
Pilot study 1: developing a ride-share intervention to improve follow-up of abnormal fecal immunochemical test (FIT) results	1. Determine critical components of a ride-share program in settings where procedural sedation is administered 2. Assess the acceptability, appropriateness, and feasibility of two ride-share models	**III**: Efficient Prototyping, RAM Test for Signal of Acceptability, Feasibility, Appropriateness	- Maximize alignment of ride-share model features with patient and provider **preferences**. - Maximize the **reach** of abnormal FIT follow-up using ridesharing.
Pilot study 2: a staged approach to implementing hereditary cancer risk assessment (HCRA)	1. Match implementation strategies to determinants to delivery of genetic testing 2. Evaluate a stakeholder-driven approach to HRCA implementation planning	**I**: Rapid Evidence Review, Determinant Prioritization **II**: CPDs	- Maximize **impact** of HCRA implementation by supporting stakeholders in matching strategies to local determinants.

*I* stage I identify and prioritize determinants, *II* stage II match strategies, *III* stage III optimize strategies.

Implementation study 1 will partner with WPRN and/or BCCHP clinics to optimize the impact of practice facilitation on colorectal cancer screening in federally qualified health centers. Practice facilitation is an effective strategy for improving preventive service delivery and chronic disease management in primary care settings [63]. However, practice facilitation can be conducted in myriad ways, resulting in varying degrees of effectiveness. This study will test (using stage III methods) whether practice facilitation’s impact on colorectal cancer screening rates can be optimized through feedback on baseline determinants (using stage I methods) and monitoring strategy-determinant alignment (using stage II methods). The study is expected to increase colorectal cancer screening rates in safety-net clinic settings and optimize a widely used, yet poorly understood implementation strategy.

Implementation study 2 will partner with KPWA to optimize strategies to increase HPV self-sampling for cervical cancer screening. In a recently completed pragmatic trial, home-based testing increased screening by 50% in a hard-to-reach population [64, 65]; however, qualitative inquiry with women who did not complete screening highlighted opportunities to optimize implementation by distributing patient-centered outreach materials with HPV self-sampling kits. Using stage II methods, the study will develop outreach materials addressing determinants specific to home-based testing and known screening determinants that might be amplified in the home-testing environment. Using stage III methods, these materials will then be “tested for signal” to ensure they address identified determinants to screening completion. These optimized outreach materials will be ready for use and evaluation in a subsequent confirmatory RCT.

Pilot study 1 will partner with Harborview Medical Center/UW Medicine to assess the acceptability, feasibility, and demand for a ride-share transportation program for patients with abnormal fecal immunochemical test (FIT). Transportation is a frequently cited determinant to colonoscopy completion and a likely contributor to lack of FIT follow-up [66]. Ride-share platforms are potentially scalable and cost-effective strategies as rides are scheduled by the health care team, costs are billed to the organization and utilization does not require individual smartphone ownership. However, ride-share programs to address this transportation determinant to screening completion can be designed in different ways; using stage III methods, this study will explore ride-share employee, patient, and provider perspectives on different ride-share program models. A ride-share program optimized for patient acceptability and demand and provider acceptability and feasibility can be tested in a subsequent trial for effectiveness in reducing disparities in follow-up of abnormal FIT.

Pilot study 2 will partner with KPWA to identify promising strategies to implement hereditary breast cancer risk assessment guidelines. Clinical guidelines recommend routine ascertainment of individuals at increased hereditary breast and ovarian cancer risk to facilitate timely access to counseling, testing, and risk-management [67]. Yet only about 20% of eligible women have ever discussed genetic testing with a health professional [68]. The study leverages an existing implementation effort in Kaiser Permanente Washington to increase access to cancer genetic services. Using stage I and stage II methods, the study will match strategies to high-priority determinants to routine hereditary cancer risk assessment and delivery of genetic testing, and evaluate a staged, stakeholder-driven approach to program implementation planning. The study will generate usable knowledge for optimizing program implementation while testing the acceptability, feasibility, and usefulness of stage II methods with stakeholders.

## Discussion

### Response to COVID-19 pandemic

The OPTICC Center began in September 2019, just before the COVID-19 pandemic. In response to public health restrictions, clinical organizations modified their operations [69]. which led to reductions in cancer care across the cancer control continuum [70–72]. In the state of Washington, where COVID-19 had the earliest impact in the USA, our I-Lab partners experienced similar changes and observed new barriers related to the pandemic. We surveyed federally qualified health centers in one of our I-Lab networks. We found that they reported substantial clinic closures and decreases in overall visits. Half reported a significant reduction in cancer screening activities, partly because staff had their time shifted to COVID-19 response. One of our other I-Lab networks analyzed Puget Sound Surveillance, Epidemiology, and End Results data immediately prior to and following the start of the pandemic impact in the US. They found that fewer cancers were being detected, and a shift to diagnosis at later stages than prior to COVID-19. Cancer patients had fewer in-office visits; some, but not all of these visits were replaced by telemedicine. Some cancer treatments, such as chemotherapy, increased during the pandemic, while other treatments, such as surgeries, declined [72]. We will interview all I-Lab partners about the impact of COVID-19 on their cancer control efforts in January 2021 to tailor our research opportunities and capacity-building efforts to their current needs.

### Health equity

As we prepare for the next group of pilot studies, the I-Lab leads will meet with representatives of each I-Lab member to learn their current cancer control priorities and implementation challenges, so that our next call for proposals can be driven largely by the implementation practice challenges faced by our partners, including those imposed by COVID-19. We will also prioritize studies that address health equity as cancer burden falls inequitably on traditionally underserved populations. To realize the Cancer Moonshot Blue Ribbon Panel’s vision [1], cancer control EBIs must be rapidly, effectively, and efficiently implemented in clinical and community settings where traditionally underserved populations receive care, work, and live. Some of the initial OPTICC studies will attempt to address health disparities by (1) testing and refining methods for optimizing EBI implementation in settings that serve racially, ethnically, and geographically diverse populations and (2) optimizing strategies that address determinants to cancer control EBIs that disadvantaged populations disproportionately experience.

### Growing a diverse workforce through measurement studies

OPTICC will also advance implementation science measurement. With funding through a diversity supplement to grow the implementation science workforce, OPTICC investigators will create a scalable, flexible method of quantitatively identifying and prioritizing determinants to EBI implementation. Focusing on the Intervention Characteristics domain of the CFIR [18]—where few reliable and valid measures exist [73]—we will develop item banks for each determinant in that domain and administer the items to a large sample of healthcare professionals in our I-Lab. Study participants will be randomly assigned one of two cancer control EBIs, which they will then rate using the items. They will also complete a measure of implementation stage and intention to use the EBI, a proximal outcome to EBI adoption. We will then use item response theory to create robust, streamlined measures that can be used to assess the intervention characteristics of a wide range of EBIs yet can be tailored to different implementation contexts. In addition, we will use multiple regression to link each determinant to implementation stage and intention to use, facilitating the development of empirically valid cut-off scores indicating whether the determinant poses a barrier or facilitator to EBI implementation.

### Dissemination

Across OPTICC-funded studies, our methods will be refined to ensure they are most efficient, economical, and useful to stakeholders. We will develop toolkits for each method and associated training opportunities in the form of MOOCs, for example. We will also create a website with separate pages for each OPTICC Center method. The front end of the website will be constructed leveraging user-centered design principles, including articulation of user archetypes and iterative design sessions. The backend of the website will contain a relational database to allow for accumulation of knowledge first within OPTICC but ideally, longer-term, across the Implementation Science Consortium and possibly beyond. The relational database will be explicitly structured around categories used in our CPDs to curate evidence across studies about determinants, strategies, mechanisms, etc., which are essentially common data elements. This information architecture will be a scientific contribution, as it will represent a way to unify and structure evidence about the operation of a wide range of diverse implementation strategies.

## Conclusions

We expect OPTICC to produce the following outcomes: (1) improved methods for identifying and prioritizing determinants, (2) refined methods for matching strategies to determinants, (3) optimized strategies ready for large-scale evaluation and use, and (4) new, reliable, valid, and pragmatic measures of key implementation constructs. In addition to changing the methods and measures used in implementation science, the Center will significantly impact public health by supporting cancer control EBI implementation, with research findings publicly available to implementers via a user-friendly website offering practical tools and guidance for optimizing EBI implementation.

## Data Availability

Not applicable.
